# Lecithin and Chitosan as Building Blocks in Anti-*Candida* Clotrimazole Nanoparticles

**DOI:** 10.3390/ph16060790

**Published:** 2023-05-25

**Authors:** Lisa Myrseth Hemmingsen, Virginia Panzacchi, Lloyd Mbugua Kangu, Barbara Giordani, Barbara Luppi, Nataša Škalko-Basnet

**Affiliations:** 1 Department of Pharmacy, University of Tromsø—The Arctic University of Norway, Universitetsvegen 57, 9037 Tromsø, Norway; virgi.panzacchi96@gmail.com (V.P.); lloyd.kangu@gmail.com (L.M.K.); natasa.skalko-basnet@uit.no (N.Š.-B.); 2 Department of Pharmacy and Biotechnology, University of Bologna, Via San Donato 19/2, 40127 Bologna, Italy; barbara.giordani4@unibo.it (B.G.); barbara.luppi@unibo.it (B.L.)

**Keywords:** chitosan, phosphatidylcholine, *C. albicans*, biocompatibility, lipid-based carriers, safety-by-design, efficacy-by-design

## Abstract

The main focus when considering treatment of non-healing and infected wounds is tied to the microbial, particularly bacterial, burden within the wound bed. However, as fungal contributions in these microbial communities become more recognized, the focus needs to be broadened, and the remaining participants in the complex wound microbiome need to be addressed in the development of new treatment strategies. In this study, lecithin/chitosan nanoparticles loaded with clotrimazole were tailored to eradicate one of the most abundant fungi in the wound environment, namely *C. albicans*. Moreover, this investigation was extended to the building blocks and their organization within the delivery system. In the evaluation of the novel nanoparticles, their compatibility with keratinocytes was confirmed. Furthermore, these biocompatible, biodegradable, and non-toxic carriers comprising clotrimazole (~189 nm, 24 mV) were evaluated for their antifungal activity through both disk diffusion and microdilution methods. It was found that the activity of clotrimazole was fully preserved upon its incorporation into this smart delivery system. These results indicate both that the novel carriers for clotrimazole could serve as a therapeutic alternative in the treatment of fungi-infected wounds and that the building blocks and their organization affect the performance of nanoparticles.

## 1. Introduction

Wounds are major burdens for the health care systems and impact the quality of patients’ lives. Chronic wounds affect about 1–2% of the general population, and it is expected that their prevalence will increase in the years to come [1]. There is a consensus that the microbial burden within the wound has great impact on healing; however, focus is mostly tied to the bacterial burden [2]. Due to the complexity of the wound microbiome, it is important to also consider other microorganisms partaking in these communities. Among the frequent participants, fungi deserve more attention [3]. In a study by Kalan et al., fungi were identified in 80% of diabetic foot ulcers examined in the study, and *Candida albicans* was found in about half of the subjects and 22% of the samples [4]. Furthermore, Dowd et al. uncovered *C. albicans* in about 46% of all fungi-positive chronic wounds [5]. In addition to their abundant presence in the wound environment, *C. albicans* partake in the microbiological community and could, together with the rest of the residents, lead to infections that further hamper the wound healing cascade [6]. Moreover, *C. albicans* could also accelerate resistance, tissue adhesion, and biofilm formation in certain bacteria, such as *Staphylococcus aureus* and *Pseudomonas aeruginosa* [7,8].

Clotrimazole (CLO) is widely used to treat topical fungal infections, especially infections that are caused by *Candida* spp. [9]. The action of this azole-type compound relies on disruption of the fungi cell wall due to inhibition of the fungal cytochrome 14α-demethylase enzyme [10]. Generally, azole-type compounds have high selectivity toward fungal cytochrome P450 enzymes as compared to the ones in mammalian cells, and therefore reduced incidence of adverse effects [11]. Nonetheless, adverse effects, such as irritation and burning sensations, although not very common, are reported [12]. Lowered water solubility, high lipophilicity, and limited bioavailability at the site of infection are among the factors limiting the current and future use of CLO in topical infection therapy [9,13,14]. However, these factors also make CLO a relevant model for antifungal compounds with similar solubilities. Although relatively potent, CLO requires a proper delivery system for effective and timely activity at the infected area. However, the current therapeutic options within antifungal therapy are jeopardized by increasing resistance pushed by over-use in agriculture and human health [15].

One of the promising approaches to circumvent or improve the limitations of CLO and other antifungal agents is tailoring lipid-based nanoparticles for delivery to the intended site of action [13]. Solid lipid nanoparticles, nanostructured lipid carriers, and liposomes with CLO have been challenged against topical fungal infections with promising results [16,17,18,19,20,21]. To further improve the antifungal potential of lipid-based nanoparticles, inclusion of chitosan could potentially improve the antifungal effects due to inherent antimicrobial actions if tailored in a smart manner [22]. Chitosan nanoparticles with natural compounds and CLO have been proven effective both in fungal eradication and wound healing [23], highlighting the potential of further exploring chitosan in topical antifungal therapy. The combinations of lipids and chitosan are highly interesting when tailoring novel delivery systems for antifungal compounds like CLO; however, the system needs to be properly designed to build on the safety and efficacy required of a formulation. Safety and efficacy by design is a common strategy to construct novel formulations [24,25]. Andersen et al. tailored chitosomes, a combination of liposomes and chitosan with metronidazole against *C. albicans* in vaginal infections [20], while Chhonker et al. designed lecithin/chitosan nanoparticles (LCNs) with amphotericin B against both *C. albicans* and *Aspergillus fumigatus* [26]. The aim was to investigate whether the building blocks and their organization could affect the anti-*Candida* properties of the model antifungal compound, CLO. LCNs were tailored based on a one-step direct injection method first described by Sonvico et al. [27] and modified by Chhonker et al. [26]. The combination of lecithin and chitosan in these hybrid nanoparticles could provide advantageous properties originating from both lipid-based systems and chitosan, such as biocompatibility, biodegradability, improved chemical stability and properties of pharmaceutical compounds, inherent antimicrobial properties, and enhanced bioavailability [28]. The novel delivery system is proposed to serve as a platform for improved treatment of fungi-infected skin wounds, especially for eradication of *C. albicans*, one of the most abundant fungi found in wound beds [4,5].

## 2. Results and Discussion

### 2.1. Nanoparticle Development

**Figure 1:**
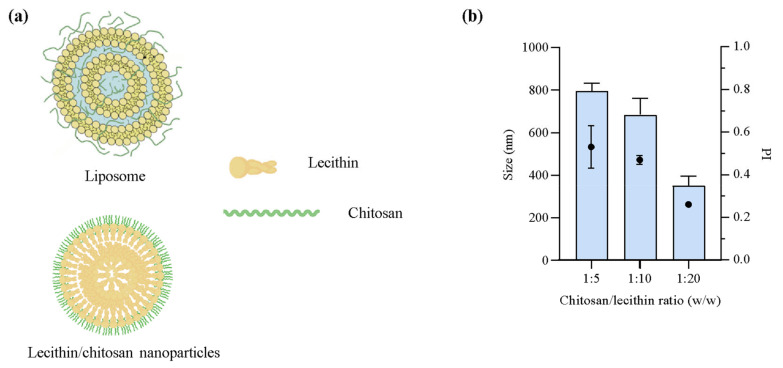
(**a**) Schematic representation of building block organization in liposomes with chitosan (top) and lecithin/chitosan nanoparticles (bottom). The drawing of the liposome is modified from Andersen et al. [20]. (**b**) Size (bars, left axis) and size distribution presented as polydispersity index (PI, symbols, right axis) of empty nanoparticles with different ratios of chitosan and lecithin. The size measurements are expressed as diameter means of cumulative size ≤75% of nanoparticle populations (weight-intensity distribution). Results are expressed as means with their respective SD (*n* = 3).

Clotrimazole is commonly used to treat fungal infections, especially in cases of candidiasis [29]. However, due to its mode of action and subsequent potential resistance development, low water solubility and bioavailability, and associated adverse or off-target effects [12,30,31], the need to improve the current therapeutic options against both local and systemic fungal infections is increasingly important [32]. Both lipid- and polymer-based nanoparticles have demonstrated promising attributes in the delivery of these antifungal compounds, which could in turn improve the therapeutic outcome of antifungal therapy [13]. Furthermore, lipid-based nanocarriers are often considered beneficial in the treatment of topical infections, especially due to their ability to improve solubility, permeability, and release performance of active compounds from the carriers [33]. These features could improve the limitations of common antifungal agents, including CLO [34]. However, the poor water solubility of CLO could also limit the selection of nanocarriers. As with other poorly water-soluble compounds, e.g., some anticancer agents, these features could limit the release from the nanocarrier and therefore also the concentration at the site of action and potentially the activity [35]. Jøraholmen et al. proved high CLO affinity for liposomes as observed through slow release from both plain liposomes and chitosan-coated liposomes [36]. Based on the results of Jøraholmen et al. [36] and the favored release of amphotericin B from LCNs in the study of Chhonker et al. [26], the LCNs were selected over liposomes as suitable carriers for CLO. The schematic representations of the organization of both liposomes and LCNs is presented in Figure 1a. To assure an appropriate size and size distribution of the LCNs, three different ratios of chitosan and lecithin were evaluated (Figure 1b).

### 2.2. Nanoparticle Characteristics

**Figure 2:**
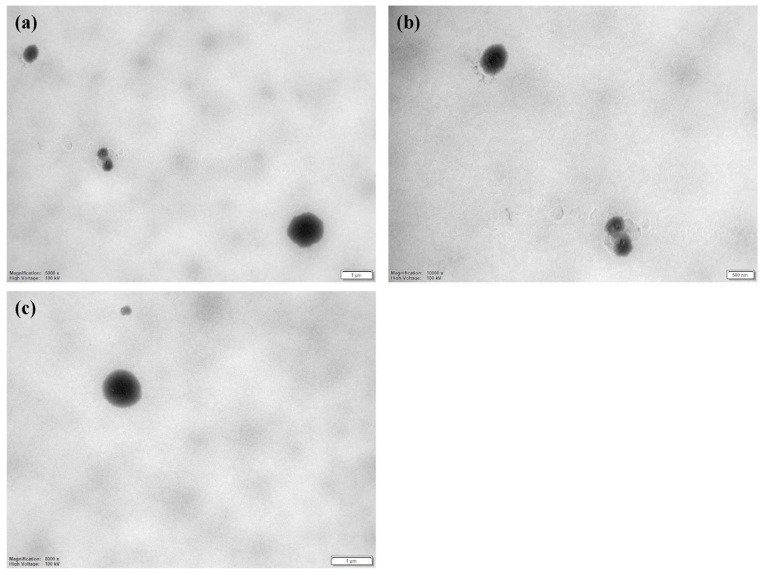
TEM images of CLO-LCNs. (**a**) Scale bar: 1 µm, (**b**) scale bar: 500 nm, (**c**) scale bar: 1 µm. CLO-LCNs = clotrimazole-lecithin/chitosan nanoparticles.

LCNs were prepared loaded with CLO in a chitosan/lecithin ratio of 1:20 to improve treatment of fungi-infected chronic wounds and therefore take advantage of the properties of both the lipid and polymer. These self-assembled nanoparticles were successfully prepared through a direct injection method, and their characteristics are presented in Table 1. The nanoparticles were also evaluated using transmission electron microscopy (TEM) as depicted in Figure 2. The cumulative average hydrodynamic diameter of 75% of nanoparticle populations was below 190 nm for the CLO-LCNs, which was significantly smaller than that of the empty LCNs (Table 1). The empty LCNs had a slightly larger size and broader size distribution than LCNs with the same lipid-to-chitosan ratio in the study by Sonvico et al. [27]. The properties of the chitosan used in the production of LCNs highly influence the size and size distribution of the nanoparticles as demonstrated by Blažević et al. [37] and Sonvico et al. [27]. Chhonker et al. evaluated chitosans of different molecular weights and observed a significant variation in size of LCNs between the different molecular weights [26]. At approximately the same molecular weight as in the current study, these LCNs were smaller; however, a lipid/chitosan ratio of 10:1 was used in the study of Chhonker et al. Both LCNs and CLO-LCNs exhibited cationic surface characteristics similar to those of the nanoparticles prepared by Blažević et al. using chitosan with similar molecular weight and degree of deacetylation [37]. The surface charge of the nanoparticles indicates that chitosan is accommodated on the surface and therefore available to interact with the fungi and assert its action. The antifungal action of chitosan is not fully elucidated; however, it is postulated that positively charged chitosan interacts with negatively charged groups on the fungal membrane, such as sialic acid [38,39]. This could give rise to stronger antifungal activity of the combination between chitosan in the LCNs and CLO. The entrapment efficiency (EE%), although not very high, is comparable to the results of Carbone et al. that reached an EE% of approximately 17% for nanostructured lipid carriers; however, these particles had a higher drug loading compared to the lipid concentrations [17]. Nonetheless, other studies reported higher EE% for various lipid-based nanoparticles [9,16,29,40]. Based on the poor water solubility of CLO that strongly contributes to its affinity for the lipophilic core of the nanoparticles, CLO was expected to accommodate itself within the lipophilic core. In a study by Clementino et al., the authors proved the accommodation of simvastatin, also a poorly water-soluble compound, within the lipophilic core of the nanoparticles [41]. Therefore, it was hypothesized that CLO is embedded within the lipophilic core of the nanoparticles.

### 2.3. Nanoparticle Stability

**Figure 3:**
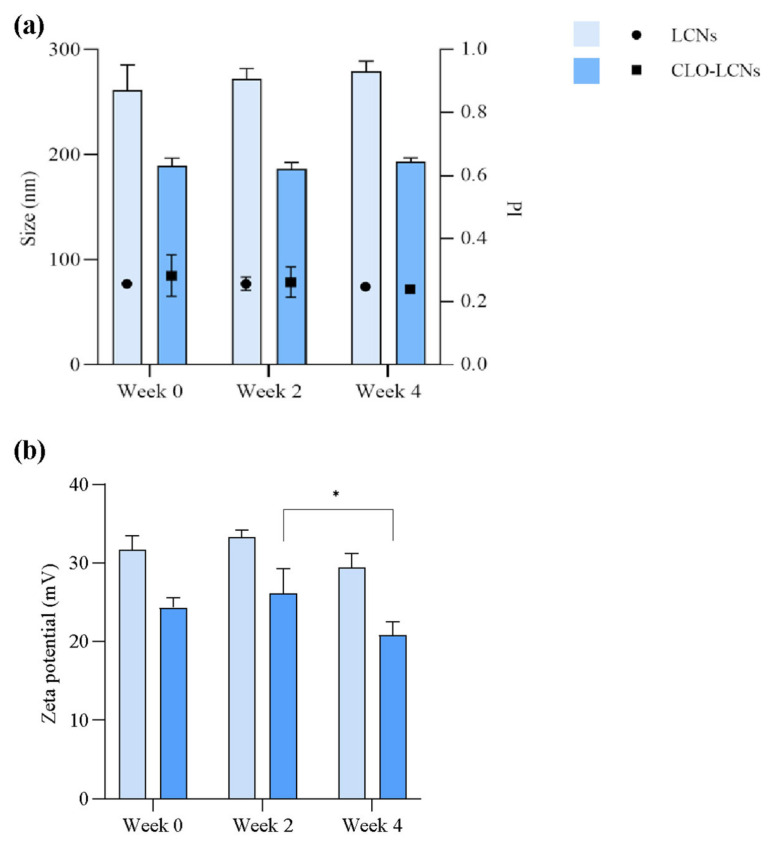
Stability of LCNs and CLO-LCNs determined as size, size distribution, and zeta potential over a period of 4 weeks. (**a**) Size is presented as total population with normal distribution (bars, left axis) with the size distribution presented as polydispersity index (PI, symbols, right axis) and (**b**) zeta potential at production and after 2 and 4 weeks (*n* = 3). LCNs = empty lecithin/chitosan nanoparticles, CLO-LCNs = clotrimazole-lecithin/chitosan nanoparticles. (*) *p* < 0.05.

In the evaluation of nanoparticle stability, it was found that both the LCNs and CLO-LCNs were relatively stable over a four-week period. No significant changes were found in size, size distribution, or surface charge between time of production and the fourth week (Figure 3). The stability of the nanoparticle formulations is often connected to the surface charge and the related repulsive effects. A surface charge above 30 mV is often considered sufficient to provide forces that are strong enough to stabilize nanoparticle suspensions [38]. There was no significant difference in the initial surface charge between the LCNs and CLO-LCNs; however, the mean surface charge of LCNs was higher than the one for CLO-LCNs (Table 1), and only the LCNs had a mean surface charge of about 30 mV. Nonetheless, both formulations were stable between the time of production and the fourth week, and therefore the stability might have also been impacted by the steric effects of chitosan. These effects have previously been demonstrated by Kurlyandskaya et al., that determined both electrostatic and steric stabilization of iron oxide nanoparticles by chitosan in water-based suspensions [42]. There was a small change in the zeta potential of the CLO-LCNs between the second and fourth week (*p* = 0.0411); however, this change was not significant between the time of production and the fourth week. Clementino et al. also investigated the stability of LCNs after up to three months of storage and found that the LCNs were stable in regard to size, zeta potential, and entrapment [41].

### 2.4. Evaluation of Toxicity

**Figure 4:**
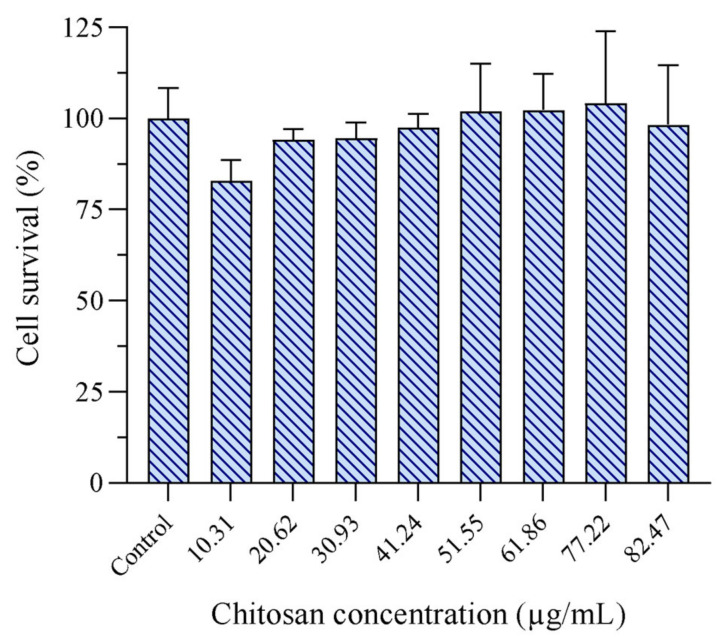
Assessment of LCN cell toxicity in HaCaT cells. The LCNs were diluted to attain chitosan concentrations ranging from 10.31 to 82.47 µg/mL; the results are presented as cell viability of treated cells compared to control (untreated cells; 100%). Control cells were supplemented with a medium; cell viability was therefore considered as 100%. The results are expressed as means with their respective SD (*n* = 3). LCNs = empty lecithin/chitosan nanoparticles.

After its discovery in the late 1960s and entry to the market in the early 1970s, CLO is widely used worldwide, especially against topical fungal infections [11,43]. It has been used locally in creams, ointments, and lotions and is known to rarely exhibit serious side effects, except from some milder responses, such as skin irritation or allergic reactions [11,44]. However, the potential toxicity of the nanoparticles or nanoparticles bearing active compounds is dependent on the constituent building blocks, preparation method, and characteristics of the nanoparticles. In the study of Blažević et al. the authors found dose-dependent toxicity of empty LCNs at the highest doses tested (20 µg/mL) in HaCaT cells [37], which raised concerns and led us to evaluate the potential keratinocytes toxicity of our LCNs using the MTT assay to investigate the cell activity. An eight-stepped concentration range from 10.31 to 82.47 µg/mL of chitosan was included. As depicted in Figure 4, no toxicity was observed in HaCaT cells treated with LCNs over the whole concentration range. Furthermore, none of the tested concentrations were significantly different from the untreated cells (*p* > 0.05). These results were more in line with the results obtained by Hafner et al., where no significant toxic effects were observed [45]. According to the ISO standard, significant toxicity is defined as ≥30% reduced cell viability [46]. Chitosan is regarded as safe, biocompatible, and biodegradable; however, it is important to investigate the potential toxicity of innovative drug delivery systems as the materials’ properties are altered in the nano-range [47]. Lecithin is also viewed as safe, especially for topical use, as it is a common ingredient in cosmetics and often used in higher concentrations than in the current study [48]. So far, lipid-associated toxicity has never been detected in our previous studies [49,50]. Manca et al. assessed the potential toxicity of CLO loaded into lipid-based vesicles in keratinocytes and found no toxicity issues in a CLO concentration range of 2 to 100 µg/mL [29]. These concentrations are far higher than the concentrations in the current study.

### 2.5. Anti-Candida Activity

**Figure 5:**
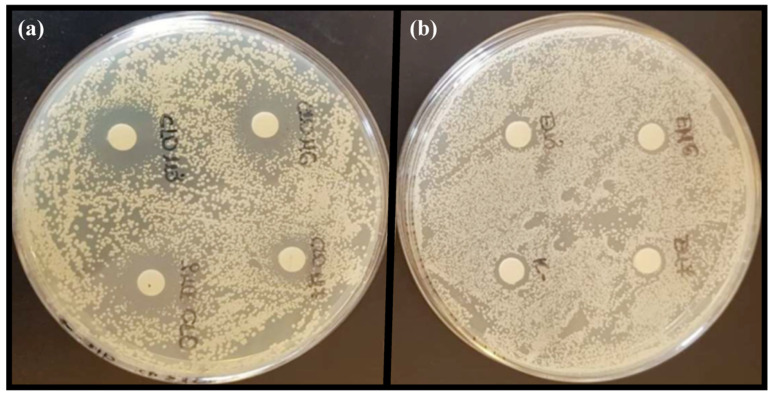
Representative images of agar plates used to determine zone of inhibition in *C. albicans*. (**a**) Free CLO and CLO-LCNs and (**b**) LCNs and control.

In order to assure that the antifungal activity of CLO was maintained in the LCNs, two different anti-*Candida* assays were performed, namely disk diffusion and broth microdilution assays. It was also evaluated whether chitosan, known for its antifungal activity [51] could enhance the activity of the CLO-LCNs. In the first assay, disk diffusion, the empty LCNs did not display any antifungal activity (Table 2 and Figure 5). On the other hand, CLO-LCNs exhibited a similar effect as free (non-formulated) CLO against both *C. albicans* SO1 and *C. albicans* SO2 (*p* = 0.43 and 0.54, respectively). From these results it was confirmed that the activity of CLO was maintained in the LCNs. Furthermore, it was noted that *C. albicans* SO2 was more sensitive to free CLO and CLO-LCNs than *C. albicans* SO1. In the study of Chhonker et al., the authors also observed similar zones of inhibition of LCN-entrapped amphotericin B and the free amphotericin B. Moreover, they demonstrated that the performance of the nanoparticles was similar to that of a marketed formulation [26].

**Figure 6:**
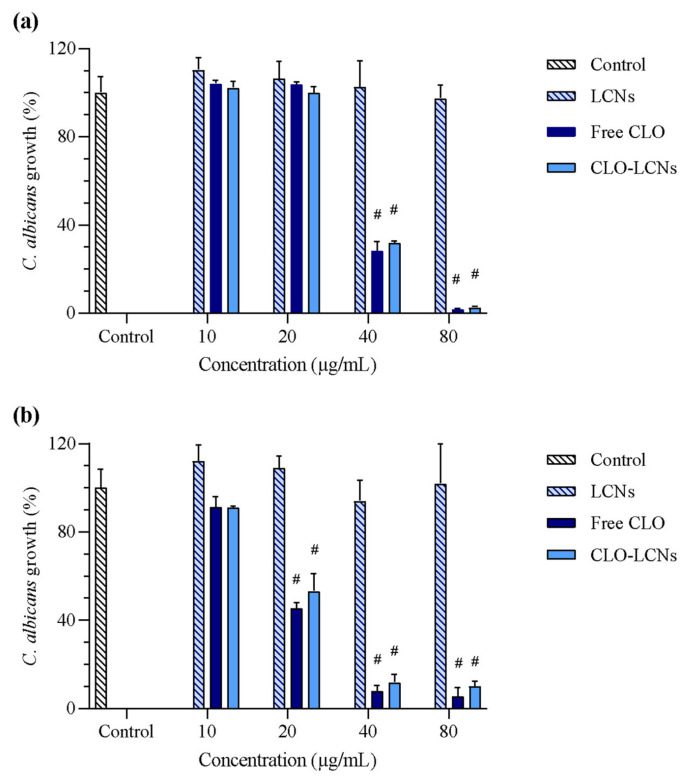
Anti-*Candida* activity assessed with the broth microdilution method. CLO concentrations ranged from 10 to 80 µg/mL; the results are presented as viability of treated fungi compared to that of control (untreated fungi; 100%). (**a**) *Candida albicans* SO1 and (**b**) *C. albicans* SO2. The results are expressed as means with their respective SD (*n* = 3). LCNs = empty lecithin/chitosan nanoparticles, free CLO = dissolved CLO (non-formulated), CLO-LCNs = clotrimazole-lecithin/chitosan nanoparticles. (#) *p* < 0.0001 compared to control.

Next, the antifungal effect of LCNs, free CLO, and CLO-LCNs was evaluated using the broth microdilution assay. In this assay (Figure 6), the results from the disk diffusion assay were confirmed. The empty LCNs did not exhibit any antifungal activity against the two *C. albicans* strains. This lack of antifungal activity from LCNs is in accordance with the results of Valencia et al. who evaluated LCNs in *C. albicans* [51,52,53]. However, both free CLO and CLO-LCNs induced a strong antifungal effect. In *C. albicans* SO1, fungi treated with free CLO or CLO-LCNs exhibited significantly reduced growth compared to that in controls at a concentration of 40 µg/mL (*p* < 0.0001) and higher. The same trend was observed in *C. albicans* SO2; however, in this case, growth was significantly reduced when the fungi were treated with free CLO or CLO-LCNs at a concentration of 20 µg/mL (*p* < 0.0001). No differences were observed between the fungi treated with CLO and CLO-LCNs, confirming that the antifungal activity of CLO was maintained in the CLO-LCNs. Chhonker et al. found similar trends in *C. albicans* treated with LCNs loaded with amphotericin B, where the antifungal activity of amphotericin B was maintained in the LCNs when assessed by a broth dilution assay. However, in the determination of the inhibition zone, the amphotericin-B-loaded LCNs produced a slightly smaller zone than the free amphotericin B did [26]. In the study of Andersen et al., the authors observed that chitosomes, chitosan-comprising vesicles, exhibited complete inhibition of *C. albicans* at chitosan concentrations of 0.11–0.22 mg/mL; however, they did not detect any additional effects of metronidazole-loaded chitosomes. Yet in their study, the proportion of lipid was far greater than that of chitosan as compared to that in the current study. Moreover, the chitosan concentrations utilized in their anti-*Candida* evaluations were also higher [20]. The antifungal activity of chitosan is highly dependent on physiochemical properties of chitosan, its accommodation in the delivery system, the structure and building blocks of the delivery system, and fungus-related factors [54]. Furthermore, chitosan is primarily expected to improve the wound-healing effect of the formulation. CLO is the main constituent responsible for eradication of *C. albicans* in the wound bed, further enabling chitosan to improve the healing. However, the capability of the CLO-LCNs to eradicate *C. albicans* was proven with the CLO concentration that was incorporated in CLO-LCNs and maintained the activity of CLO. For that reason, the CLO-LCNs were not optimized further to improve CLO entrapment. The results reported in Figure 6 clearly revealed that CLO exhibited a dose-responsive effect towards both *Candida* isolates. In particular, the growth of *C. albicans* SO1 was strongly impaired up to concentration of 40 µg/mL, whilst the growth of *C. albicans* SO2 was reduced by about half even at the concentration of 20 µg/mL.

## 3. Materials and Methods

### 3.1. Materials

Chitopharm™ M—Chitosan with medium molecular weight (average of 350–600 kDa) and degree of deacetylation of >70% was kindly provided by Chitinor (Tromsø, Norway). Lipoid S45 was kindly provided by Lipoid GmbH (Ludwigshafen, Germany). Methanol ≥99.9%, HiPerSolv CHROMANORM^®^ for LC-MS, acetonitrile HiPerSolv CHROMANORM^®^ gradient for HPLC (≥99.9%), ethanol HiPerSolv CHROMANORM^®^ gradient for HPLC (96% *v*/*v*), hydrochloric acid, and glycerol AnalaR NORMAPUR^®^ (86%) were purchased from VWR International (Fontenay-sous-Bois, France). Acetic acid (≥99.8%), RPMI-1640 Medium, with L-glutamine and sodium bicarbonate, Thiazolyl Blue Tetrazolium Bromide, powder, BioReagent, suitable for cell culture, ≥97.5% (HPLC, MTT), Trypan Blue solution, 0.4%, liquid, sterile-filtered, suitable for cell culture, Trypsin-EDTA solution, 0.25%, sterile-filtered, BioReagent, suitable for cell culture, fetal bovine serum (FBS), and Dulbecco’s Phosphate Buffered Saline modified, without calcium chloride and magnesium chloride, pH 7.4, suitable for cell culture, were acquired from Sigma-Aldrich (St. Louis, MO, USA). Clotrimazole, potassium phosphate monobasic (≥99.0%), propylene glycol (≥99.5%, FCC, FG), sodium chloride (puriss. p.a., ≥99.5% AT), isopropanol, and sodium phosphate dibasic dehydrate (purum p.a., crystallized, ≥99.0% T) were purchased from Sigma-Aldrich (Steinheim, Germany). Triton X-100, for molecular biology, Merck KGaA (Darmstadt, Germany). Dimethyl sulfoxide (DMSO, 99.5%) analytical reagent A.R., Lab-Scan Ltd. Analytical Sciences (Dublin, Ireland). EMS Uranyless EM stain, Electron Microscopy Sciences (Hatfield, England). Sabouraud dextrose broth Difco™ (final pH 5.6 ± 0.2), Becton, Dickinson & Company (Le Pont de Claix, France). HaCaT cell line (immortalized human keratinocytes) was purchased from AddexBio (San Diego, CA, USA) [55]. *C. albicans* SO1 and *C. albicans* SO2 were from Professor Vitali’s culture collection, University of Bologna (Bologna, Italy) [56].

### 3.2. Nanoparticle Preparation

The preparation of LCNs and CLO-LCNs were based on a method described by Sonvico et al. with minor modifications [27]. A chitosan solution (1%, *w*/*v*) was prepared in acetic acid (1%, *w*/*v*), stirred for 1 h, and left to rest overnight. The chitosan solution (0.5 mL) was then diluted with 44 mL milli-Q water. An ethanolic lecithin solution (2.5%, *w*/*v*, 4 mL) with CLO (final theoretical concentration: 0.82 mg/mL) was injected into the diluted chitosan solution (2 mL/min), stirred at 450 rpm for 1 h at 24 ± 1 °C, and stored at 4 °C. Empty LCNs were prepared in the same manner without CLO. Furthermore, different ratios of chitosan and lecithin were evaluated in the empty LCNs, namely 1:5, 1:10, and 1:20, respectively. These concentration ratios of chitosan and lecithin were selected based on previous work on this type of LCNs [26,27,37,45]. Furthermore, the concentration of CLO was selected and adjusted to the reported activity in the product monography of marketed formulations comprising CLO, the CLO release profiles reported for other lipid-based systems [36], and consideration that the LCNs need a secondary vehicle to be administered to the skin. The compositions of the selected LCN formulations for further assessments are defined in Table 3.

Unentrapped CLO was separated from the CLO-LCNs by centrifugation. The CLO-LNCs were centrifuged with a Beckman L8-70M Ultracentrifuge with a Beckman SW 60 Ti rotor (Beckman Coulter Inc., Palo Alto, CA, USA) at 3000x *g* for 30 min at 10 °C. CLO was quantified by reversed-phase HPLC using a Waters e2795 separations module combined with a Waters 2489 UV–VIS detector (Waters Corporation, Milford, CT, USA). The separation was carried out using a Waters X Terra™ RP_18_ column (5 µm, 3.9 × 150 mm) and a Waters X Terra™ RP_18_ guard cartridge (5 µm, 3.9 × 20 mm, Waters Corporation, Milford, CT, USA). The mobile phases consisted of acetonitrile and milli-Q water, and the gradient started at 30% acetonitrile with an increase to 70% over 10 min and to 100% after 13 min. The flow rate, injection volume, and detection wavelength were set to 1 mL/min, 20 µL, and 210 nm, respectively. The procedure was modified from a method by Jøraholmen et al. [36].

### 3.3. Characterisation of Nanoparticles

#### 3.3.1. Size, Zeta Potential, and pH Determination

The nanoparticle diameter was determined using a NICOMP Submicron particle sizer (NICOMP Particle Sizing System, Santa Barbara, CA, USA) according to a previously described method [57]. Briefly, the nanoparticle suspensions were diluted with filtered (0.2 µm) distilled water to attain an intensity of 250 to 350 kHz. The diluted suspensions were measured in four cycles of 10 min, and the weight-intensity distribution was recorded.

The zeta potential was measured using a Zetasizer Nano Zen 2600 (Malvern, Worcestershire, UK). The nanoparticle suspensions were diluted (75 µL in 1 mL) in filtered (0.2 µm) tap water to ensure counter ions according to the attenuation, and measured in three cycles at 25 °C [57].

pH was measured with an Accumet^®^ portable pH meter AP115 (Fisher Scientific, Waltham, MA, USA) at room temperature (24 ± 1 °C) for all the nanoparticle suspensions. The nanoparticles were measured in their initial state, dispersed in milli-Q water.

#### 3.3.2. CLO-LCN Morphology

The morphology of the CLO-LNCs was determined using TEM. The CLO-LNCs were deposited on glow discharged 200–400 mesh carbon-coated grids for 5 min, stained with uranyless for 10–40 s, and air-dried for 30 min. The images were acquired with an HT7800 Series transmission electron microscope (Hitachi High-Tech Corp., Tokyo, Japan) running at an accelerated voltage of 100 kV connected to a Morada camera.

#### 3.3.3. Nanoparticle Stability

Nanoparticle stability was evaluated by measuring the size and zeta potential after 2 and 4 weeks of storage at 4 °C as described in Section 3.3.1.

### 3.4. Toxicity Evaluation

The potential toxicity of the LCNs was evaluated in HaCaT cells using an MTT assay as previously described [58]. In short, cell suspensions (2 × 10^5^ cells/mL) in RPMI were seeded (100 µL) in 96-well plates and incubated overnight at 37 °C with 5% CO_2_. The cells were treated with LCN suspensions at chitosan concentrations of 10.31, 20.62, 30.93, 41.24, 51.55, 61.68, 77.22, and 82.47 µg/mL and incubated for four hours (37 °C with 5% CO_2_). Triton (2%, *v*/*v*) and RPMI served as positive and negative controls, respectively. MTT (10 µL, 5 mg/mL in phosphate-buffered saline) was then added to each well, and the plates were incubated for 2 h (37 °C with 5% CO_2_). After incubation, 70 µL of the medium was removed from each well, and after 100 µL of 0.04 M HCl in isopropanol was added, the plates were placed on an orbital shaker for 1 h at 24 °C. Absorbance was measured at 590 nm. Cell survival of the treated cells was compared to that of the control.

### 3.5. Anti-Candida Evaluation

*C. albicans* SO1 and SO2 were grown aerobically on Sabouraud Dextrose agar plates for 24 h at 30 °C. The cells were suspended and diluted in a Sabouraud Dextrose broth to reach a concentration of 2 × 10^6^ CFU/mL to create the inoculum for anti-*Candida* testing.

#### 3.5.1. Anti-*Candida* Activity Determined as Zone of Inhibition

In the first evaluation of anti-*Candida* activity, the disk-agar diffusion method [59] was utilized to evaluate the activity of LCNs, CLO-LCNs, and non-formulated CLO (dissolved in DMSO, 160 µg/mL). A solvent control containing the same amount of DMSO as in the non-formulated CLO solution was also prepared and included. The inocula of the test organisms (100 µL, 10^6^ CFU/mL) were spread on Sabouraud Dextrose agar plates (9 cm diameter). Sterile cotton disks (6 mm in diameter) were impregnated with 20 µL of each sample and placed on the surface of the plates. The plates were kept at 4 °C for 1 h to allow the diffusion and subsequently incubated aerobically for 48 h at 30 °C. The zone of inhibition was determined using an electronic digital caliper.

#### 3.5.2. Anti-*Candida* Activity Determined through Broth Microdilution

The second method used to evaluate anti-*Candida* activity was broth microdilution [21]. The LCNs, CLO-LCNs, and CLO in DMSO (1 mg/mL) were two-fold serial diluted in distilled water in 96-well plates to concentrations between 10 and 80 µg/mL. The cell suspensions were inoculated in 96-well plates together with 50 µL of the diluted samples. Yeast suspension inoculated together with distilled water served as growth control, and growth medium and distilled water served as sterility controls. The plates were incubated aerobically for 24 h at 30 °C. Anti-*Candida* activity was determined by comparing the turbidity (OD_600_) of growth control with treated cells using an EnSpire Multimode Plate Reader (PerkinElmer Inc., Waltham, MA, USA).

### 3.6. Statistical Analyses

The results are generally expressed as the mean ± SD. Student’s *t*-tests or one-way ANOVA with Tukey’s post-test were performed to evaluate significance (*p* ≤ 0.05) using GraphPad Prism version 9.3.1 for Windows (GraphPad Software LLC, San Diego, CA, USA).

## 4. Conclusions

The results of the current study indicate that CLO-LCNs could serve as delivery systems for topical delivery of CLO to fungi-infected wounds due to their safety and efficacy profiles. Although the entrapment efficacy of CLO was not high, CLO retained its activity that was sufficient to induce the desired antifungal effect. It was also proposed that the organization of lecithin and chitosan as building blocks affects the activity of the nanocarrier.

## Figures and Tables

**Table 1:** Nanoparticle characteristics—size, size distribution, surface charge, entrapment efficiency, and pH of LCNs and CLO-LCNs.

	Size ^1^ (≥75%, nm)	PI ^2^	Zeta Potential (mV)	EE% ^3^	pH
LCNs	350 ± 47	0.26 ± 0.01	31.7 ± 4.0	-	4.19 ± 0.07
CLO-LCNs	189 ± 24	0.28 ± 0.07	24.3 ± 4.0	14.7 ± 2.5	4.39 ± 0.11

Results are expressed as means with their respective SD (*n* = 3). LCNs = empty lecithin/chitosan nanoparticles, CLO-LCNs = clotrimazole-lecithin/chitosan nanoparticles. ^1^ The size measurements are expressed as diameter means of cumulative size ≤75% of nanoparticle populations (weight-intensity distribution). ^2^ Polydispersity index. ^3^ Entrapment efficiency (%).

**Table 2:** Anti-*Candida* activity assessed as zone of inhibition (mm) in *Candida albicans* SO1 and SO2.

	Zone of Inhibition		
	LCNs	Free CLO	CLO-LCNs
*C. albicans* SO1	N.I.	4.12 ± 0.37	3.83 ± 0.44
*C. albicans* SO2	N.I.	7.06 ± 0.57	6.79 ± 0.40

Results are expressed as means with their respective SD (*n* = 3). LCNs = empty lecithin/chitosan nanoparticles, free CLO = dissolved CLO (non-formulated), CLO-LCNs = clotrimazole-lecithin/chitosan nanoparticles, N.I. = no inhibition.

**Table 3:** Composition of selected LCNs and CLO-LCNs.

	LCN	CLO-LCNs
Chitosan/lecithin ratio	1:20	1:20
CLO	-	0.82 mg/mL

CLO = clotrimazole, CLO-LCNs = clotrimazole-lecithin/chitosan nanoparticles, LCNs = empty lecithin/chitosan nanoparticles.

## Data Availability

Data are contained within the article.
